# Antibody Avidity Maturation Following Booster Vaccination with an Intranasal Adenovirus Salnavac Vaccine

**DOI:** 10.3390/vaccines12121362

**Published:** 2024-12-02

**Authors:** Ekaterina A. Astakhova, Konstantin O. Baranov, Nadezhda V. Shilova, Svetlana M. Polyakova, Evgeniy V. Zuev, Dmitry A. Poteryaev, Alexander V. Taranin, Alexander V. Filatov

**Affiliations:** 1 Laboratory of Immunochemistry, National Research Center Institute of Immunology, Federal Medical Biological Agency of Russia, 115522 Moscow, Russia; avfilat@yandex.ru; 2 Department of Immunology, Faculty of Biology, Lomonosov Moscow State University, 119234 Moscow, Russia; 3 Moscow Center for Advanced Studies, Kulakova Street 20, 123592 Moscow, Russia; 4 Laboratory of Immunogenetics, Institute of Molecular and Cellular Biology, Siberian Branch of the Russian Academy of Sciences, 630090 Novosibirsk, Russiataranin@mcb.nsc.ru (A.V.T.); 5 Shemyakin and Ovchinnikov Institute of Bioorganic Chemistry, Russian Academy of Sciences, 117997 Moscow, Russia; 6 JSC “GENERIUM”, 12311 Moscow, Russiapoteryaev@ibcgenerium.ru (D.A.P.)

**Keywords:** avidity, antibody maturation, SARS-CoV-2, COVID-19, Salnavac vaccine, Sputnik V vaccine, biolayer interferometry

## Abstract

Background: The COVID-19 pandemic has led to the rapid development of new vaccines and methods of testing vaccine-induced immunity. Despite the extensive research that has been conducted on the level of specific antibodies, less attention has been paid to studying the avidity of these antibodies. The avidity of serum antibodies is associated with a vaccine showing high effectiveness and reflects the process of affinity maturation. In the context of vaccines against SARS-CoV-2, only a limited number of studies have investigated the avidity of antibodies, often solely focusing on the wild-type virus following vaccination. This study provides new insights into the avidity of serum antibodies following adenovirus-based boosters. We focused on the effects of an intranasal Salnavac booster, which is compared, using a single analytical platform, to an intramuscular Sputnik V. Methods: The avidity of RBD-specific IgGs and IgAs was investigated through ELISA using urea and biolayer interferometry. Results: The results demonstrated the similar avidities of serum antibodies, which were induced by both vaccines for six months post-booster. However, an increase in antibody avidity was observed for the wild-type and Delta variants, but not for the BA.4/5 variant. Conclusions: Collectively, our data provide the insights into antibody avidity maturation after the adenovirus-based vaccines against SARS-CoV-2.

## 1. Introduction

The induction of a humoral immune response to a pathogen is a crucial goal of vaccination. The effectiveness of a vaccine is thought to be strongly associated with high titers of the specific antibodies produced [1,2,3]. However, the quality of the humoral immune response is also defined by the avidity of serum antibodies, reflecting the process of affinity maturation in the lymph nodes’ germinal centers [4,5]. Avidity accounts for the accumulated affinities of monoclonal antibodies in a serum, reflecting the strength of their bi- or multivalent bindings with epitopes [6]. High-affinity antibodies optimize antiviral functions, including virus neutralization and antibody-dependent cell-mediated cytotoxicity, and they promote immune complex formation [6,7,8]. Despite the importance of antibody affinity, this parameter has not been studied to the same extent as the level of specific antibodies. Some studies have established the increase in avidity index (AI) values induced by the HIV-1, rubella, mumps, Dengue, and influenza virus vaccines [9,10,11,12,13]. The prevalence of low-affinity antibodies is associated with severe Zika virus infection, influenza disease, and COVID-19 [14,15,16,17], as well as a high risk of repeated reinfection with some seasonal coronaviruses [18]. As for anti-SARS-CoV-2 immunity, a minority of studies have included data on avidity, with only some measuring avidity with regard to the wild-type virus following anti-SARS-CoV-2 vaccinations [19,20,21,22,23].

The COVID-19 pandemic opened up a new era characterized by the rapid development and approval of new vaccines [24]. Since the emergence of the Omicron variant family in 2021 and its evasion of pre-existing immunity, there has been widespread discussion about new approaches that could be used to induce a more robust humoral response. The most well-known approaches include vaccine strain adaptation to new variants of concern (VOCs) and the adjustment of the regimens used for booster administration [23,25,26,27]. However, another promising approach involves altering the administration route of the vaccine from intramuscular to mucosal, as the mucous membranes of the respiratory tract serve as the entry point for the SARS-CoV-2 pathogen [28]. The main expectation regarding mucosal vaccines against COVID-19 is the induction of mucosal immunity, such as the production of secretory IgA and tissue-resident T and B cells in the upper and lower respiratory tracts [29]. Several studies have demonstrated that both mucosal and systemic immunity are induced by intranasal vaccines against COVID-19 [30,31,32,33]. This immunity, however, is weaker than that induced by intramuscular boosters [34]. Here, we were interested in how large this difference was in the specific case of the Salnavac vaccine. Moreover, there are no or little data on the avidity of serum antibodies after intranasal vaccination [35,36]. The goal of the present study was to better understand the affinity maturation of serum antibodies induced by intranasal vaccination.

Salnavac is an adenovirus vector-based vaccine and is similar to well-known vaccines such as AstraZeneca, Johnson & Johnson, CanSino, and Sputnik V [37,38,39]. However, unlike the latter, it is administered intranasally [32]. In this study, we compared SARS-CoV-2 antibody avidities following boosters with intramuscular Sputnik V and intranasal Salnavac. We monitored the affinity maturation of RBD-specific IgGs and IgAs using both chaotrope-based avidity-ELISA and chaotrope-free biolayer interferometry (BLI). We determined AIs for both wild-type (WT) RBD and the Delta and BA.4/5 variants, providing new insights into vaccine-induced avidity to VOCs compared with previous studies [40,41,42]. Therefore, we demonstrate that antibody avidity to the WT and Delta variants, but not to BA.4/5 variants, increases six months after the boosters.

## 2. Materials and Methods

### 2.1. Ethics

The study protocol was approved by the Russian Ministry of Health (No. 869 of 20 December 2021) and the independent local ethical committees of the clinical centers. The study was conducted in accordance with the requirements of Good Clinical Practice and the World Medical Association Declaration of Helsinki (revision 2013). All of the volunteers provided signed informed consent before being included in the study.

### 2.2. Volunteers

Eighty-five healthy volunteers were included in this study. The main inclusion criteria were (1) an age of 18–60 years; (2) a level of initial anti-RBD-IgG antibodies to SARS-CoV-2 no higher than 100 BAU/mL; and (3) the absence of an acute infectious or non-infectious disease and the absence of the exacerbation of a chronic disease 14 days before immunization. The main exclusion criteria were (1) vaccination against SARS-CoV-2 within a period starting six months before inclusion in the study; (2) a history of COVID-19 six months before inclusion; (3) a positive PCR test or IgM result for SARS-CoV-2 at screening; (4) the use of steroid drugs (except contraceptives); (5) the use of immunoglobulins and/or other blood components 30 days before inclusion, or the use of immunosuppressants three months before inclusion; (6) difficulty in nasal breathing; (7) the regular use of decongestants; and (8) the presence of concomitant diseases affecting the results of the study.

This study included the screening of volunteers boosted with two components of the adenovirus-based vaccine Sputnik V or Salnavac and an observation period (up to six months). The median ages of the volunteers were 31 (range 23–60 years) and 34 (range 20–58) for Sputnik V- and Salnavac-boosted patients, respectively. Both groups were similar overall in terms of the male/female ratio (23/22 male and 22/18 female in the groups of Sputnik-V-/Salnavac-boosted donors, respectively).

### 2.3. Vaccines

Two vaccines were used: Sputnik V and Salnavac. Salnavac is an intranasal variant of the previously authorized Sputnik V vaccine [32].

Both vaccines comprised two components: a recombinant viral vector based on human adenovirus serotype 26 (Ad26, component I) and serotype 5 (Ad5, component II) with the full-length virus S-protein gene SARS-CoV-2. The full dose of both vaccines is 1011 viral particles per dose for each recombinant adenovirus or 0.5 mL per dose for intramuscular/intranasal new introduction. As a placebo, a buffer composition equal to the volume of vaccine was used.

### 2.4. SARS-CoV-2 Pseudovirus Production

Lentiviral particles pseudotyped with the SARS-CoV-2 S protein of the WT strain were obtained as previously described [32]. We used a mixture of three plasmids for transfection HEK293T cells (a kind gift from Dmitriy Mazurov, Institute of Gene Biology Russian Academy of Sciences, Moscow, Russia): psPAX2, reporter NanoLuc, and a pCAGGS-SpikeΔ19 plasmid. The virus-like particles (VLPs) were aliquoted and stored at −70 °C. The titration of VLPs was performed on HEK293T-ACE2 cells. The optimal dosage of VLPs was determined as the number of VLPs required to obtain 1000 relative units (RUs) in the luciferase test.

### 2.5. SARS-CoV-2 S-Pseudotyped Lentivirus Neutralization Assay (pVNA)

To provide pVNA, 15,000 HEK293T-ACE2 cells per well of a 96-well plate were seeded one day before each test. Sera were serially diluted in Opti-Mem (Gibco, Waltham, MA, USA) + 2.5% heat-inactivated FBS in twofold steps (range 1:10–1:1280) and co-incubated with VLP for 30 min at 37 °C in a volume of 180 µL. The cell culture medium was replaced by HEK293T-ACE2, and 130 µL of sera and a VLP mixture were added to HEK293T-ACE2 cells. Two days after incubation at 37 °C, the 5% CO_2_ culture media were replaced, and the cells were lysed with PBS + 0.5% Triton X100 for 5 min with a plate rotator (500 rpm) at room temperature. Then, the luciferase substrate OneGlo (Promega, Madison, WI, USA, dilution 1:100) was added, and luciferase activity was measured using a Luminoskan Microplate (Thermo Fisher Scientific, Waltham, MA, USA). The half-maximal inhibitory dilution (ID_50_) was determined by fitting a sigmoidal curve (GraphPad Prism 9.2.0 software, Sigmoidal, 5PL) reconstructed by the percentage of neutralization at the different indicated serum dilutions.

### 2.6. Source of Recombinant Proteins

Recombinant RBD-His6 proteins (WT, Delta, and Omicron BA.4/5) were expressed and purified as previously described [43]. The pCAGGS-SΔ19 plasmid, encoding the S protein, included the following mutations: T19R, G142D, Δ156–157, R158G, L452R, T478K, D614G, P681R, D950N (Delta); T19I, Δ24–26, A27S, Δ69–70, G142D, V213G, G339D, S371F, S373P, S375F, T376A, D405N, R408S, K417N, N440K, L452R, S477N, T478K, E484A, F486V, Q498R, N501Y, Y505H, D614G, H655Y, N679K, P681H, N764K, D796Y, Q954H, and N969K (Omicron BA.4/5).

### 2.7. Biolayer Interferometry Measurement of K_D_, k_on_, and k_off_ for Serum Antibodies

The kinetic parameters of interaction between sera and RBD were determined by biolayer interferometry using ForteBio Octet RED96 (Sartorius, Göttingen, Germany). Ni^2+^-NTA sensors bound to the 6× His-tag present on the antigen. Recombinant RBD proteins and irrelevant protein with similar M_w_ were diluted in assay buffer (PBS, 0.05% Tween20, 0.05% NaN_3_) in a concentration of 8 μg/mL. Serum samples were also diluted in assay buffer at 1:30. Only one serum dilution was chosen for k_off_s determination using BLI because it was previously shown that the k_off_ rate did not depend on the specific antibody concentration [13]. One Ni^2+^-NTA biosensor was used for sera from three timepoints from one donor. One cycle of measurement for the kinetic parameters of one sample included the following steps: (1) The biosensor was soaked in assay buffer, and the first baseline was measured for 180 s. (2) The antigen was loaded onto the sensors for 300 s. (3) The biosensor was soaked in assay buffer, and the second baseline was measured for 120 s. (4) Serum antibodies were associated with the antigen for 400 s and then (5) dissociated for 600 s when the sensors were soaked again in assay buffer. The regeneration step, followed by each cycle, included immersing the sensors three times in glycine (pH 1.7) and kinetic buffer for 5 s and then immersing them in 10 mM NiCl_2_ for 60 s. For each sample, the kinetics of the irrelevant protein were subtracted from the RBD kinetics to reduce non-specific binding signals. K_off_ measurement was based on the Full Global Fitting, Bivalent model, R^2^ > 0.9, Χ^2^ < 3 using Octet Red96 operating software (version 9.0.0.10; FortéBio, Göttingen, Germany).

### 2.8. Avidity ELISA

For the avidity ELISA, 96-well plates (Immulon 4 HBX; Thermo Scientific, Waltham, MA, USA) were coated overnight at 4 °C with 100 µL/well of recombinant RBD proteins (1 µg/mL) in phosphate-buffered saline. The plates were washed three times with PBS supplemented with 0.05% Tween-20 (Sigma-Aldrich, Darmstadt, Germany) between each step of incubation. Then, 150 µL of blocking buffer (Xema Co., Moscow, Russia, Cat. No. S010) was added to each well and left for 2 h at room temperature (RT). Next, the serum samples, diluted 1:30 with sample buffer (Xema Co., Cat. No. S011), were incubated for 1 h at RT in duplicate. For IgG RBW WT avidity, ELISA serial dilutions were performed (1:10, 1:30, 1:90, and 1:270). Thereafter, the same sample was incubated in side-by-side wells, and one well was treated with 100 µL/well of 8M urea (diluted with PBS) while the other was treated with the same volume of PBS. The plates were incubated for 30 min at RT. After washing, the plates were incubated with 50 µL/well of anti-human IgG or IgA antibody (Jackson Immuno Research, West Grove, PA, USA, Cat. No. 109-036-088 and 109-035-011, respectively) conjugates with horseradish peroxidase (HRP), diluted 1:5000 with buffer for conjugates (Xema Co., Cat. No. S012). After 1 h of incubation and washing, 100 µL/well of TMB chromogen solution (Xema Co.) was added and left for 20 min. The reaction was stopped by adding 50 μL/well of 1M H_2_SO_4_. The optical density (OD) at 450/520 nm was measured using a Synergy 4 (BioTek, Thermo Scientific, Waltham, MA, USA) plate reader.

The avidity index (AI) was calculated using the following formula:

AI = OD (urea-treated sample)/OD (non-treated sample).

An ELISA (not an avidity ELISA) was performed as described above but excluding the step of incubation with urea or PBS. One sample was incubated on each plate and was used as a plate-to-plate control. The results of ELISA were recorded in relative units (RUs) that corresponded to OD with plate-to-plate correction.

### 2.9. Surrogate Virus Neutralization Assay on Chips (sVNT)

Chips were printed on epoxy-activated slides from Semiglass Epoxy (Semiotik LLC, Moscow, Russia) by the robotic arrayer SciFlexArrayer S5 (Scienion, Berlin, Germany), with a drop volume of 1 nL. One grid included RBD WT, RBD Delta, RBD BA.4/5, and immunoglobulin G (IgG, Sigma)-Alexa488 as the fluorescence control; the BSA solution in the print buffer (see below) and block and print buffers served as negative controls. The concentration of each protein was 100 µg/mL. The print buffer consisted of 300 mM phosphate buffer containing 0.001% Tween20. One array contained six replicates of each ligand and was used for one sample. One chip contained six arrays: five of them were incubated with serum samples in step two (see below) and one was incubated with the sample buffer. After printing, the chips were incubated in the chamber at 75% relative humidity and 37 °C for protein immobilization. The fluorescent intensities (as RFUs, relative fluorescent units) of each RBD variant obtained from this array indicated the maximum level of binding RBD with fluorescent-labeled ACE2.

The chips were kept in a blocking buffer (consisting of 50 mM ethanolamine (Sigma-Aldrich, Darmstadt, Germany), 100 mM boric acid (Lumi, Moscow, Russia), and 0.2% Tween-20 (pH 8.5)) for two hours under continuous stirring at room temperature, and they were washed three times with phosphate-buffered saline containing 0.05% Tween-20 after each step. In the second step, the serum samples, which were diluted 1:30 with the sample buffer (PBS + 0.1% BSA + 0.1% Tween 20 + 0.001% NaN_3_), were applied to a chip and incubated for 1 h at 75% relative humidity. Then, ACE2 labeled with Alexa488 (Hytest Co., Moscow, Russia, Cat. No. 8AE5) diluted 1:3500 in the sample buffer was added to the chip, and after 30 min incubation at a relative humidity of 75%, the chips were finally washed with bidistilled water, dried, and scanned with an InnoScan 1100 AL fluorescence reader (Innopsys, Carbonne, France) at 10 µm resolution. The images were analyzed with ScanArray Express 4.0 software using the fixed-circle method (PerkinElmer, Waltham, MA, USA). The median relative fluorescence units (RFUs) indicated the level of binding of ACE2 with the RBD proteins. To calculate a neutralization index (sVNT titer), we used the following formula:

(1 − (RFU (sample + ACE2-Alexa488) − b)/(RFU (ACE2-Alexa488 only) − b)) ×100%,

where “b” is the background fluorescence signal of the chip (RFU value of the ligand-free surface).

### 2.10. Statistical Analysis

Statistical analysis of the significance of differences between groups was performed in GraphPad Prism 9.2.0, with non-parametric analogous ANOVA (the Kruskal–Wallis multiple-comparison test) for the BLI and pVNA and two-way ANOVA (Sidak’s multiple-comparison test) for the ELISA, avidity assay, and sVNT.

## 3. Results

### 3.1. The Avidity of RBD-Specific mAbs

Prior to the analysis of the avidities of vaccine-induced sera, we investigated the correlation between the different affinity parameters of RBD-specific monoclonal antibodies. For this, we used two independent methods: biolayer interferometry (BLI) and avidity ELISA. BLI determines the kinetic characteristics of antigen–antibody interactions, while avidity ELISA is an equilibrium-based assay.

**Figure 1:**
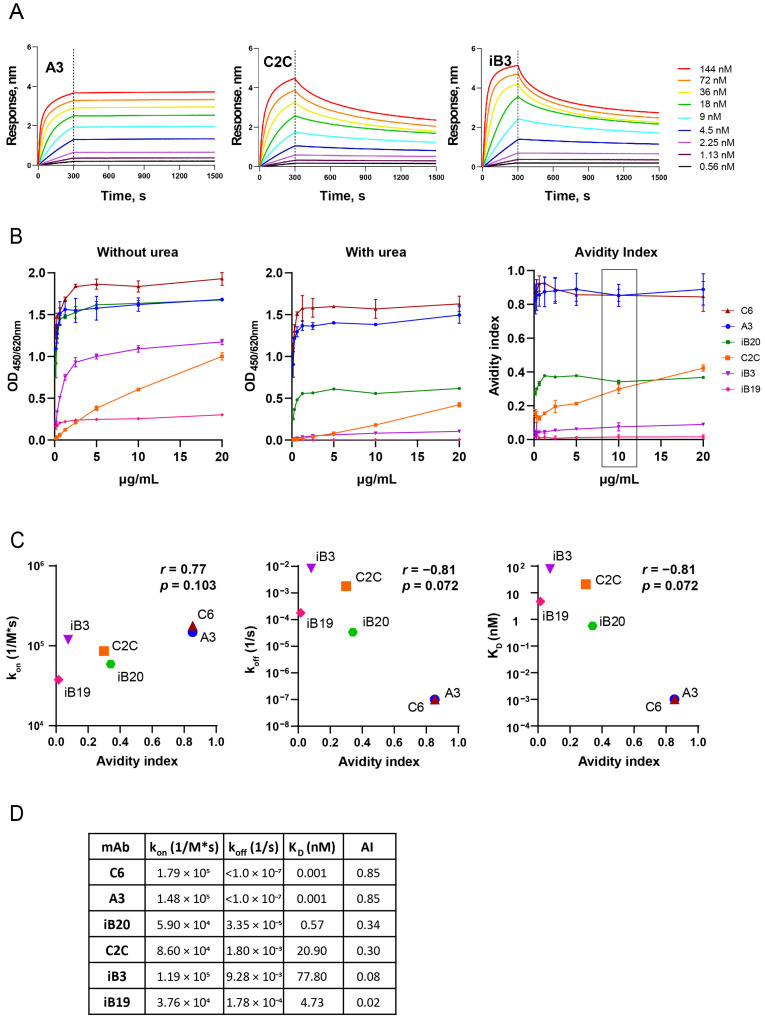
Affinity of anti-RBD monoclonal IgG antibodies. (**A**) Representative sensorgrams for mAbs with high (**left** panel), medium (**middle** panel), and low (**right** panel) affinity. The dotted line indicates the time between the association and dissociation steps. (**B**) The RBD (WT) binding capacity of mAbs with (**middle**) or without (**left**) 8 M urea treatment is shown. Dependence affinity indexes for the mAb concentration (**right**) are also shown. The means of the triplicate measurements and SD are presented. The frame indicates the chosen concentration of mAbs to determine AIs. (**C**) Spearman’s correlation between the affinity indexes of mAbs and k_on_, k_off_, and K_D_. (**D**) Summary table of the mAb affinity parameters.

The binding affinity to RBD (WT) was tested for 6 IgG human monoclonal antibodies neutralizing SARS-CoV-2 that we had previously generated [43]. To measure the binding kinetics of antibody-to-antigen interaction, the sensors immobilized with His-tagged RBD WT were transferred to wells containing assay buffer and a known concentration of mAbs. Representative sensorgrams for A3, C2C, and iB3, as high-, medium-, and low-affinity mAbs, respectively, are presented in Figure 1A. The 6 mAbs tested differed in K_D_ by approximately five orders of magnitude and ranged from 0.001 to 77.80 nM (Figure 1B). K_D_ 0.001 nM is the limit of detection for OctetRed96.

Next, for each mAbs, ELISA binding profiles were obtained with recombinant wild-type RBD both in the presence of urea and without it (Figure 1B).

In untreated samples, ELISA signals reached a plateau as the concentration of mAb increased (Figure 1B, left panel). The amount of RBD-bound IgG decreased after the exposure of immune complexes to 8M urea, and the dilution curves of urea-treated samples looked similar to treated samples, but with lower plateau levels (Figure 1B, middle panel).

Next, we determine the avidity of mAbs based on the ELISA results. Technically, the term “affinity” is the most common for mAbs versus “avidity”, even though they are bivalent and the proper term for them is, in fact, “avidity” [44]. To prevent confusion between the terms that are used for monoclonal antibodies and polyclonal sera later on, we introduced the common term “avidity index” (AI) for both, and we provide a formula for AI in the Methods section.

The avidity indexes (AIs) were calculated for each concentration of mAbs from 0.1 to 20 μg/mL (Figure 1B, right panel). AIs for all mAbs except C2C were similar across mAb dilutions in the concentration range of 5 to 20 μg/mL. In contrast, the AI of C2C showed a linear dependence on the mAb concentration. MAbs with greater affinity demonstrated increased resistance to 8M urea treatment. The mAbs C6 and A3, which had the lowest K_D_, retained at least 85% of their binding activity to SARS-CoV-2 RBD, while the antibodies with the highest K_D_ values (iB3 and iB19), in the presence of 8M urea, almost completely lost the ability to bind to the antigen.

To compare the avidity ELISA results and BLI assay, Spearman’s rank correlation analysis was performed. In this analysis, we used AI values obtained at a concentration of 10 μg/mL. We found that the AI strongly and negatively correlated both with k_off_ (*r* = −0.81, *p* = 0.072) and K_D_ (*r* = −0.81, *p* = 0.072). A weaker positive correlation between AI and k_on_ was also observed (*r* = 0.77, *p* = 0.103) (Figure 1C).

A summary of the kinetic parameters and AIs of the six tested mAbs is presented in Figure 1D.

### 3.2. Study Design and Blood Sampling

The study of avidity maturation was a part of a randomized, double-blind, multicenter, phase 3 clinical trial with a “double-dummy” design conducted from February 2022 to October 2023 to evaluate the immunogenicity of the intranasal and intramuscular forms of the combined vector vaccine (Ad26/Ad5-based) against SARS-CoV-2. For this study, 85 participants were randomly chosen from the clinical trial cohort after “unblinding”. All of the participants received a prime full Sputnik V vaccination six or more months before the present study. Individuals from the core cohort (*n* = 40) received Salnavac by intranasal injection and the placebo by intramuscular injection. In the comparison cohort, individuals (*n* = 45) received Sputnik V by intramuscular injection and the placebo by intranasal injection. The second (Ad5-based) booster was administered 21 days after the first (Ad26-based) booster.

**Figure 2:**
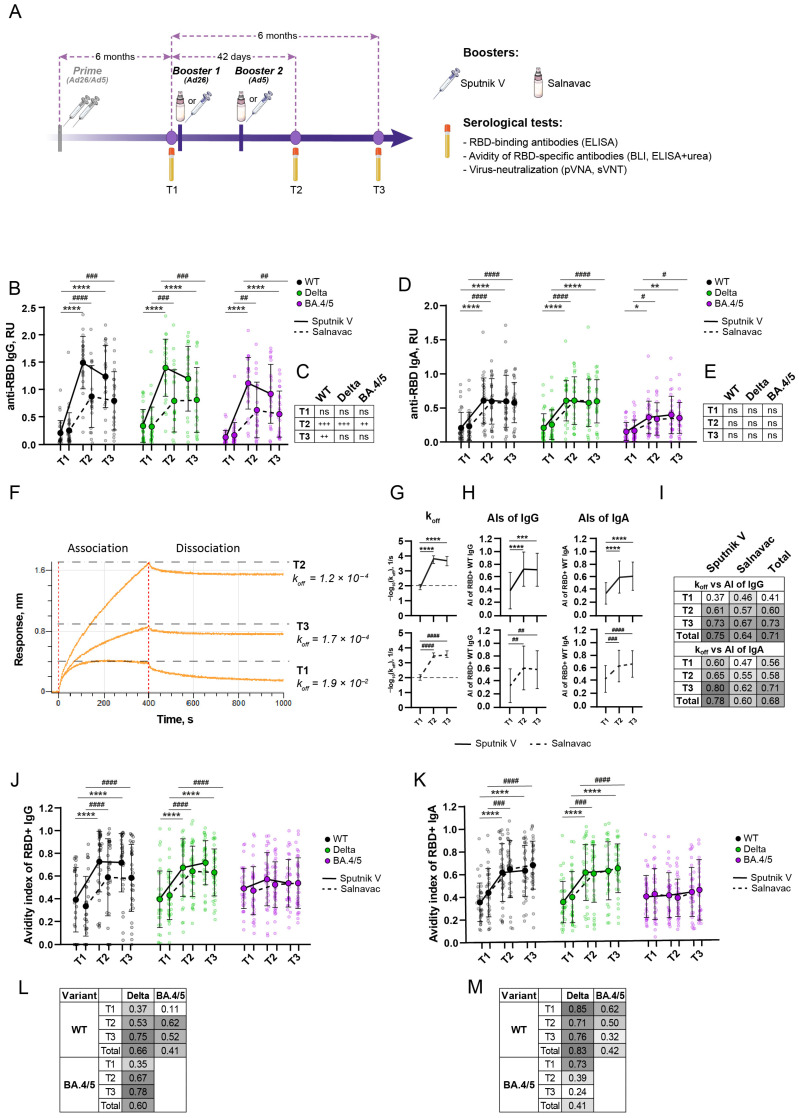
The RBD binding and avidity of serum antibodies in the boosted individuals. (**A**) Vaccination regimen, sampling timepoints, and serological tests. (**B**) Anti-RBD (WT, Delta, and BA.4/5) IgG responses in sera from Sputnik V- or Salnavac-boosted individuals. (**C**) Comparative analysis of anti-RBD IgG levels in individuals boosted with Sputnik V and Salnavac. The number of “plus” symbols or “ns” corresponds to the *p*-value as described below. (**D**) Anti-RBD (WT, Delta, and BA.4/5) IgA responses in sera from individuals boosted with Sputnik V or Salnavac. (**E**) Comparative analysis of anti-RBD IgA levels in individuals boosted with Sputnik V and Salnavac. (**F**) Representative sensorgrams of sera from one donor at the T1, T2, and T3 timepoints. The red dotted line indicates the time between the association and dissociation steps. (**G**) k_off_s of serum samples over time obtained from Sputnik V- (**top**) and Salnavac-boosted (**bottom**) individuals. (**H**) Temporal changes in the AIs of RBD-specific IgG and IgA serum antibodies from Sputnik V- (**top**) and Salnavac-boosted (**bottom**) individuals. (**I**) Spearman’s correlation between the avidity indexes of sera and k_off_ values at different timepoints. (**J**,**K**) Avidity indexes of IgG (**J**) and IgA (**K**) RBD (WT, Delta, and BA.4/5)-specific serum antibodies from boosted individuals. (**L**,**M**) Spearman’s correlation between levels of IgG (**L**) and IgA (**M**) RBD-specific serum antibodies and avidity indexes of sera and k_off_ values at different timepoints. Statistics were calculated using 2-way ANOVA with Sidak’s multiple-comparison test (**B**–**E**,**J**,**K**) and the Kruskal–Wallis test with Dunn’s multiple-comparison test (**G**,**H**). Asterisks and hashes indicate the differences between different timepoints in Sputnik V- and Salnavac-boosted individuals, respectively. Pluses (**C**) indicate differences between Sputnik V- and Salnavac-boosted donors. * (#) *p* < 0.05; ** (##, ++) *p* < 0.01; *** (###, +++) *p* < 0.001; **** (####) *p* < 0.0001; ns—non-significant. In (**B**,**D**,**J**,**K**), half-transparent dots indicate individual values, solid dots indicate the mean values, and bars are presented as SD. In (**G**,**H**), the median ± IQRs are presented.

Serum samples were collected at the following timepoints: T1—before booster 1, T2—42 days after booster 1, and T3—six months after the booster (Figure 2A).

### 3.3. A Strong Anti-RBD Humoral Immune Response Lasts at Least Six Months After a Booster with Sputnik V/Salnavac

We first examined the impact of the boosters on the level of serum antibodies against three RBD variants: WT, Delta, and Omicron BA.4/5. The latest Omicron variants were not included in this study because WT-based vaccines induce a low humoral response to them. Intranasal vaccines are known to induce a mucosal-specific IgA response [45], but their effect on the systemic IgA response has been less studied. Because Salnavac is an intranasal vaccine, we included an evaluation of RBD-specific IgA alongside the standard evaluation of RBD-specific IgG.

The levels of IgG and IgA serum antibodies against each studied variant showed a strong increase one month after the booster dose for both vaccines (Figure 2B,D). The fold changes between T1 and T2 for IgG antibodies were 7/3, 5/2, 9/4 for RBD WT, Delta, and BA.4/5, respectively (Sputnik V/Salnavac, Figure 2B). Intriguingly, the most significant increase in IgG reactivity was noted against BA.4/5, which represents the most antigenically distinct variant from the vaccine strain. At T3, the levels of serum anti-RBD IgG and IgA antibodies for all of the variants studied remained elevated compared with T1, but they showed no difference from T2. This means that a high level of specific antibodies is maintained for a minimum of six months after the Salnavac or Sputnik V booster.

Next, we compared the Sputnik V and Salnavac boosters. At T2, individuals who received the Sputnik V booster showed elevated levels of anti-RBD IgG antibodies in comparison to those who received the Salnavac booster (*p* = 0.0003, 0.0002, 0.004 for WT, Delta, and BA.4/5, respectively) (Figure 2C). At T3, the level of anti-RBD IgG antibodies from Sputnik V-boosted individuals was higher than the level from Salnavac-boosted individuals only for WT (*p* = 0.021).

The fold changes between T1 and T2 for anti-RBD IgA antibodies were lower than for IgG and were consistent for both vaccines: 3, 3, 2 for WT, Delta, BA.4/5, respectively (Figure 2D). In contrast to IgG antibodies, the fold change in anti-RBD (BA.4/5) antibodies was the lowest. In addition to IgG, at T3, the level of RBD-specific IgA antibodies was significantly higher than at T1, but it did not differ from T2.

Despite the fact that Salnavac is an intranasal vaccine, the level of anti-RBD IgA antibodies from Salnavac-boosted donors was not higher than the level from Sputnik V-boosted donors at all the timepoints for all of the studied variants (Figure 2E).

### 3.4. Avidity Indexes of Polyclonal Sera Measured by BLI and ELISA with Urea Show High Concordance

In the next step, we measured the avidity of polyclonal serum antibodies.

Both Salnavac and Sputnik V are based on the ancestral Spike and, therefore, the main measurements were performed using RBD WT. First of all, serum avidity to WT RBD was measured using BLI. In contrast to K_D_ and k_on_, k_off_ can be determined by BLI without knowing the specific antibody level in a serum [46]. K_off_ was deduced from the analysis of the dissociation curves of sera from the RBD WT-coated biosensor. Lower k_off_ shows stronger interactions between antibodies and RBD, which correspond to more avid antibodies.

The representative sensorgrams for T1, T2, and T3 are shown in Figure 2F. Slower dissociation kinetics are noticeable in the serum samples at timepoints T2 and T3 compared to T1 (Figure 2F).

The dynamics of serum antibody avidity maturation was similar for both vaccines (Figure 2G). Therefore, k_off_ increased significantly one month after the administration of the vaccine at T2 and then remained at the same level for following six months (T3). The k_off_ median values were 9.5 × 10^−3^, 3.5 × 10^−4^, and 3.02 × 10^−4^ for the Salnavac-boosted individuals and 1.3 × 10^−2^, 1.6 × 10^−4^, and 2.1 × 10^−4^ for the Sputnik V-boosted individuals (*p* < 0.0001, T1 vs. T2, and T1 vs. T3 for both vaccines). At T2, the sera from individuals who received the Sputnik V booster exhibited significantly higher avidity levels than those of Salnavac-boosted (*p* = 0.0002) donors. However, there was no difference at the timepoints T1 and T3.

We should emphasize that the results obtained by BLI in this study reflect the avidity level of total RBD-specific antibodies. The separate determination of IgG and IgA avidity by BLI is quite difficult due to technical limitations. The level of specific anti-RBD IgG antibodies was too low in the total IgG antibodies to be detected using anti-human IgG biosensors (IHC sensors). Anti-human IgA biosensors are not currently commercially available. To reveal the contribution of the IgG and IgA antibodies separately, we performed urea ELISA.

As noted previously, the choice of antibody and chaotropic agent concentrations is critical in the reliable estimation of antibody avidity [47]. To determine the optimal serum dilution in preliminary experiments, we determined the AIs of the serum samples for the RBD WT at serum dilutions from 1:10 to 1:270 (Supplementary Figure S1A).

At T2 and T3, for most sera (39/45 and 29/40 for Sputnik V- and Salnavac-boosted individuals), the AIs changed slightly over the dilution range studied. However, at T1, most sera (31/45 and 32/40 for Sputnik V- and Salnavac-boosted individuals) AIs dropped significantly upon dilution and tended to reach zero values at a dilution of 1:270. Based on this, 1:90 was chosen as a compromise dilution for the AIs of RBD WT-specific IgGs.

The variations in the AIs of the serum samples for the RBD WT exhibited a comparable trend to the k_off_ detected by BLI analysis. Therefore, both vaccines induced increases in AIs from T1 to T2 (*p* < 0.0001, for both IgG and IgA) and then, at T3, AIs remained as high as at T2 but were significantly higher than at T1 (*p* < 0.0001, for Sputnik V-boosted individuals, and *p* < 0.01 for Salnavac-boosted individuals) (Figure 2H). There was a difference between the AIs of Sputnik V- and Salnavac-boosted donors at the T2 only at the serum dilution of 1:90 (*p* = 0.046, Supplementary Figure S1B).

In addition to monoclonal antibodies, we also observed a strong correlation between the avidity levels obtained by BLI and IgG ELISA with urea (*r* = 0.71, *p* < 0.0001) (Figure 2I). The correlation for the BLI and IgA ELISA results was relatively high (*r* = 0.68, *p* < 0.0001), but it was lower than for the BLI and IgG ELISA results. In fact, these correlations indirectly reflect the contribution of IgG and IgA avidity to the whole avidity of the sera measured by BLI.

### 3.5. Booster Vaccination Induced an Increase in Avidity Index for WT and Delta RBD but Not BA.4/5 RBD

An important indicator of vaccine-induced antibodies is the breadth of their virus-binding and virus-neutralizing activity. We decided to check how much the avidity maturation that we determined for RBD WT applied to other mutant variants. We sought to determine the breadth of avidity.

The avidity indexes of the serum samples for RBD variants were measured by ELISA with urea (Figure 2J,K).

Both IgG and IgA serum antibodies, one month after the booster, became more avid to WT and Delta (T1 vs. T2; *p* < 0.0001 for AIs of IgG antibodies, both boosters; *p* < 0.0001 and *p* < 0.001 for AIs of IgA antibodies for Sputnik V- and Salnavac-boosted participants, respectively).

The AIs of RBD-specific IgG increased from T1 to T2 in 1.8/1.8 (WT) and 1.5/1.7 (Delta) times in the group of individuals boosted with Salnavac/Sputnik V, respectively. In contrast to the level of anti-RBD antibodies, no differences in the AIs were observed between the two vaccines at T2. The AIs of RBD-specific IgA increased 1.5/1.7 times for WT and Delta (Salnavac/Sputnik V, respectively).

Six months after the booster, the AIs of WT and Delta RBD-specific antibodies did not change; they remained at the level of T2 and were significantly higher than at T1 (*p* < 0.0001 for both vaccines, both IgG and IgA antibodies, T1 vs. T3).

The boosters with both vaccines did not affect the AIs in relation to BA.4/5. The AIs of anti-BA.4/5 RBD antibodies remained at a fairly low level, 0.5 and 0.4 for IgG and IgA, respectively, at all timepoints. This means that less than 50% of the serum antibodies were resistant to urea treatment.

Finally, we compared the avidity indexes for RBD WT and the variants (Figure 2L,M). For the IgG antibodies, we observed a relatively high correlation between the AIs for WT vs. Delta (*r* = 0.66, *p* < 0.0001) and the AIs for Delta vs. BA.4/5 (*r* = 0.6, *p* < 0.0001) (Figure 2L). This means that the serum samples with higher AIs for WT (Delta) tended to also show high AIs for Delta (BA.4/5). Interestingly, the correlation coefficients between AIs (WT vs. Delta) and AIs (Delta vs. BA.4/5) increased from T1 to T3 (*r* = 0.37, 0.53, 0.75 and *r* = 0.35, 0.67, 0.78, respectively). The correlation between IgG antibodies specific to antigenically distinct variants (RBD WT and BA.4/5) was weak *(r* = 0.41, *p* < 0.0001).

A strong correlation between the AIs of IgA antibodies was observed only when comparing anti-RBD (WT) with anti-RBD (Delta) antibodies (*r* = 0.83, *p* < 0.0001) (Figure 2M). The correlation coefficients between AIs for WT and Delta vs. BA.4/5 were weak (*r* = 0.42, 0.41, respectively). Moreover, Spearman’s *r*-values decreased from T1 to T3 (*r* = 0.62, 0.5, 0.32 for WT vs. BA.4/5, and *r* = 0.73, 0.39, 0.24 for Delta vs. BA.4/5, values correspond to T1, T2, and T3, respectively). The breadth of RBD-specific IgA antibodies appears to decline over the six months following the booster, unlike the IgG-specific antibodies.

### 3.6. The Relationship Between the Serum Neutralizing Activity and Avidity After Sputnik and Salnavac Booster Vaccination

Next, we looked into the functional consequences of enhanced antibody avidity. The serum neutralizing activity was evaluated by a pseudovirus-based (pVNA) virus neutralization assay.

**Figure 3:**
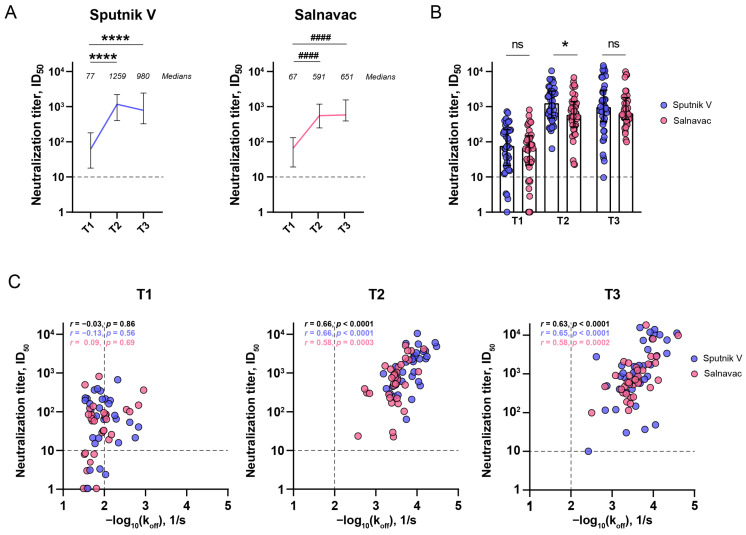
Comparative analysis of the avidity and neutralizing capacity of the sera from boosted individuals. (**A**) Neutralization antibody titers (ID_50_) against SARS-CoV-2 (WT) pseudoviral particles before and after Sputnik V (left) and Salnavac (right) booster vaccination as measured by pVNA. (**B**) Comparisons of neutralizing antibody titers between Sputnik V- and Salnavac-boosted individuals. (**C**) Spearman’s correlations between the k_off_ values and neutralization titers (pVNA) of boosted donors’ sera at different timepoints. Statistics were calculated using the Kruskal–Wallis test with Dunn’s multiple-comparison test (**A**,**B**). * *p* < 0.05; **** (####) *p* < 0.0001; ns—non-significant. In (**A**,**B**), the median ± IQR are presented. In (**B**,**C**), dots indicate individual values.

The pre-booster level of ID_50_ obtained by pVNA at T1 was above the baseline measured for pre-pandemic samples (Figure 3A). This indicates that most Sputnik V-vaccinated individuals maintain elevated levels of neutralizing antibodies against WT pseudoviral particles for up to six months following the primary vaccination. Both boosters induced an increase in neutralization titers against wild-type SARS-CoV-2 pseudoviral particles (*p* < 0.0001, T1 vs. T2). The neutralizing capacity of sera was significantly higher at T3 than at T1 (*p* < 0.0001), but it did not differ from T2 for either booster vaccine. The median titers for Sputnik V-boosted donors were 77, 1259, and 980, and for Salnavac-boosted donors, they were 67, 591, and 651 (T1, T2, and T3, respectively).

There was a significant difference in the median values of ID_50_ between the Sputnik V- and Salnavac-boosted samples at T2 (*p* = 0.043) (Figure 3B).

Next, we defined the correlations between the neutralizing activity of sera and avidity (Figure 3C). The neutralization titers obtained by pVNA with WT pseudoviruses were compared to k_off_ obtained by BLI. We observed a positive correlation between the k_off_ of RBD (WT)-specific antibodies and the level of virus neutralization expressed in ID_50_ at T2 and T3 (*r* = 0.66 and *r* = 0.63, respectively, *p* < 0.0001 for both) but no correlation between these parameters at T1 (*r* = 0.09, *p* = 0.69).

### 3.7. Surrogate Chip-Based Virus Neutralization Test

To test the virus neutralizing activity of serum antibodies against variants, we developed the rapid chip-based method (surrogate virus neutralization test, sVNT). The principle of this method is the competitive binding of fluorescently labeled ACE2 and serum antibodies with an RBD-coated chip. This approach is convenient in that, for one serum sample, it is possible to simultaneously determine the neutralization titers against several variants of the virus.

**Figure 4:**
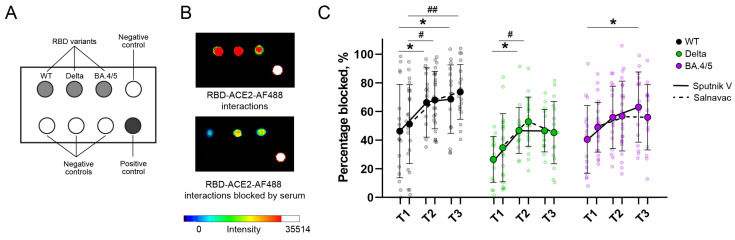
Surrogate chip-based virus neutralization test on sera from Sputnik V- and Salnavac-boosted individuals. (**A**) The chip’s print layout for the virus neutralization test. The chip was printed with RBD (WT, Delta, and BA.4/5), irrelevant protein (BSA), or buffers (print, block, or PBS) as the negative control, and AlexaFluor-488 labeled IgG as the positive control. (**B**) Representative photographs of the chips after RBD-ACE2 interaction with (low panel) and without (upper panel) the blocking of the serum. (**C**) Comparisons of neutralization measured by sVNT between Sputnik V- and Salnavac-boosted individuals. Statistics were calculated using 2-way ANOVA with Sidak’s multiple-comparison test (**C**). Asterisks and hashes indicate differences between different timepoints in the Sputnik V- and Salnavac-boosted groups of donors, respectively. * (#) *p* < 0.05; ## *p* < 0.01. In (**C**), half-transparent dots indicate individual values, solid dots indicate the mean values, and bars are presented as SD.

The level of interaction of AlexaFluor488-tagged ACE2 with the RBD spot, expressed in RFUs, was considered 100% binding. Sera blocked the interaction between ACE2 and RBD, reducing the fluorescent intensity of wells (Figure 4). The neutralization activity (sVNT titers) corresponded to the percentage of blocked ACE2-RBD interactions.

At T1, the sVNT titers for Delta and BA.4/5 were negligibly diminished relative to WT (the mean values for sVNT titers were 46/51, 27/35, and 41/49 for WT, Delta, and BA.4/5 in Sputnik V-/Salnavac-boosted donors, respectively) (Figure 4C). Both vaccines led to a notable 1.5-fold rise in neutralization against the WT and Delta variants at T2 in comparison to T1 (*p* < 0.05). A high level of neutralizing antibodies (NAbs) against the WT strain was sustained for up to six months following the booster dose (*p* < 0.05, *p* < 0.01; T1 vs. T3 for Salnavac and Sputnik V, respectively). We also observed a 1.6-fold increase in vaccine-induced neutralizing antibodies against the BA.4/5 variant after Sputnik V booster vaccination (*p* < 0.05, T1 vs. T3). sVNT WT, Delta, and BA.4/5 RBD titers did not depend on the booster at any of the timepoints for any of the studied variants (Figure 4C).

## 4. Discussion

In this study, we compared the maturation of serum antibody avidity to SARS-CoV-2 RBD over a period of six months after receiving muscular and intranasal Ad-vector-based vaccines, i.e., Sputnik V and Salnavac, respectively. Studies on the humoral immune response through COVID-19 vaccination largely focus on assessing the binding and neutralization titers. These titers depend on the concentration of specific antibodies and their affinity. Unlike the concentration of antibodies, the antibody affinity after vaccination against COVID-19 has not been widely studied. An increasing antibody affinity over time is a key characteristic of the humoral immune response that is associated with stronger neutralizing activity and is crucial for optimal vaccine efficacy [48]. There has not been a comprehensive analysis of the influence of the route of injection on the quality of systemic-induced antibodies performed on a single analytical platform.

Avidity assay studies are important in order to reveal the potential immune complex-mediated deleterious effects of vaccine-induced antibodies [13]. The avidity of serum antibodies is directly or indirectly related to the process of antibody maturation in the germinal centers of lymph nodes and B-cell memory formation [4]. It is of current interest because for some vaccines, the quality and quantity of antibodies are excellent, but the memory is low [49].

SPR, BLI, and other chaotrope-free methods enable the determination of kinetic parameters such as K_D_, k_on_, and k_off_, and were traditionally used to describe the affinity characteristics of mAbs [50]. The existing math models describe well the immune complex formation between mAb and antigens, and, therefore, their interaction can be readily calculated. The binding of polyclonal antibodies to antigens is a heterogeneous process that is difficult to describe correctly. First of all, only k_off_, not K_D_ or k_on_, can be measured, because the relevant concentration of specific antibodies in serum is unknown [50]. Furthermore, models that consider the contribution of different antibody k_off_ rates on the observed dissociation time courses of pAbs-antigen interactions are desirable. Such models are essential in gaining a deeper understanding of the diversity in pAbs avidity [51]. Therefore, Kan Li et al. developed a polyclonal antibody avidity resolution tool (PAART) to define the avidity of pAbs [51].

The easiest and most widespread method of assessing the avidity index (AI) of serum samples is to perform ELISA in the presence of chaotropic agents such as urea, thiocyanate, or MgCl_2_ [15,52,53,54]. In such conditions, low-affinity antibodies are eluted and the number of chaotrope-resistant antibodies reflects the sample avidity [11]. Although this method is thought to be the “gold standard” due to its simplicity, speed, and low cost, the protocol for this assay needs to be standardized [55] because the serum and chaotropic agent concentrations, time, and temperature of incubation, as well as other parameters, often vary and have an influence on the measured AIs [54]. Using polyclonal Abs (pAbs), Dimitrov et al. showed that antibody concentration is a crucial parameter in AI assessment [47]. Therefore, residual binding with antigens differs when a relatively low pAbs concentration is used, and residual binding is stable at relatively high pAbs concentrations. Our results with six human anti-RBD IgG mAbs are in concordance with this.

It is known that the direct adsorption of some antigens onto the polystyrene surfaces of ELISA plates can induce conformational changes and the distortion of antigen epitopes [56]. Generally, this effect only occurs in a subset of the sorbed antigen molecules, while others retain their functional activity [57,58]. A more important drawback of chaotrope-based assays is the potential partial denaturation of the quaternary structural epitopes or unfolding under chaotrope agent incubation [9]. In such conditions, AI does not reflect the functional affinity of target antibodies.

The interactions between the AI and kinetic parameters obtained with BLI or SPR are unclear. For example, M. Alexander, studying HIV-1 envelope glycoproteins, showed that AI did not correlate well with functional affinity, as described by K_D_ [9]. In the present study, we revealed a good correlation between urea-treated ELISA and chaotrope-free-BLI for mAbs (Figure 1B). Due to this, we hypothesize that in our test system, the impact of the direct adsorption of RBD onto polystyrene surfaces on its antigenic properties is not significant. Moreover, the chaotrope predominantly disrupted antibody–antigen interactions and not the structural integrity of the epitope. In addition, the k_off_s of the serum samples from Sputnik V- or Salnavac-boosted donors correlated well with the AIs of RBD (WT)-specific antibodies (the correlation for IgG was slightly better than for IgA) (Figure 2I). However, we observed statistically significant differences in the k_off_s between Sputnik V- and Salnavac-boosted serum samples at T2 (Figure 2G), whereas the IgG AIs of these samples at T2 differed only at the 1:90 serum dilution. Based on these results, we suggest that BLI is a more sensitive method than ELISA with urea, and we highlight the potential advantage of the BLI method in determination serum avidity. Currently, the applications of SPR or BLI assays for complex clinical specimens are limited.

Six months after the booster, there were no differences in avidity between the two studied groups of donors. Therefore, we showed that intranasal vaccination induced as high an avidity level in serum antibodies to RBD (WT) as an intramuscular vaccination.

The other aspect that we studied was avidity maturation to RBD variants. Prophylactic vaccination against COVID-19 via repeated administration of the same vaccine based on WT has been implemented in many countries for a long time and is still applied in some places today. Recently, for influenza vaccines, this schedule was shown to possibly adversely affect the affinity maturation [44,59,60] and lead to a reduction in the breadth of neutralization. This question is currently being actively discussed. Some studies show reduced neutralizing activity to VOCs after repeated boosters with WT (Wuhan)-based vaccines [61,62,63]. Others show improved antibody diversity, but with much lower orders of magnitude for VOC neutralization compared to WT [64,65]. Following the “breadth of neutralization” studies, we investigated the “breadth of antibody avidity”, which we define as the presence of high-avidity antibodies for several viral variants. Studying the breadth of antibody avidity is important in guiding vaccination strategies against COVID-19. High-avidity antibodies in sera indirectly show that the B cells that produce these Abs were successfully selected in germinal centers. In this study, we showed that booster vaccination with WT-based vaccines (rAd-based) leads to an increase in avidity to WT and Delta RBD, but not BA.4/5 RBD, during the six months after vaccine administration. This suggests that booster vaccination did not result in an increase in plasma B cells secreting high-avidity antibodies to BA.4/5. A low level of avid anti-BA.4/5 antibodies results in reduced efficacy in neutralizing BA.4/5, aligning with previous research highlighting the central role of high-avidity antibodies in neutralizing SARS-CoV-2 [66].

This study developed a surrogate virus neutralizing test (sVNT) on chips that we applied to sera from boosted donors. sVNTs are based on competitive binding inhibition and reveal the ability of serum RBD-specific antibodies to block the interaction of ACE2 with RBD or S-protein [67]. This test is attractive because it does not require work with cells or live viruses. Some studies have shown a high correlation between sVNTs and conventional virus neutralizing tests. Finally, sVNTs may be automatized and used in large screening assays [67].

sVNTs had already been present at the beginning of the COVID-19 pandemic [68], enabling the detection of neutralizing nAbs for WT RBD or variants, but in a single-well format [69,70,71]. The emergence of multiple SARS-CoV-2 variants has increased the need for multiplexing platforms to effectively assess neutralizing activity against these variants. Kara Lynch et al. have developed a multiplexed sVNT reaction that utilizes spike proteins coupled to spectrally distinct paramagnetic beads [72]. The microarray-based format of sVNT has shown potential in detecting neutralizing activity to WT, early variants, Delta, and Omicron [73,74,75].

In contrast to traditional tests that involve serial dilutions of sera, sVNTs typically utilize a single dilution of the sample. This is a serious limitation in the detection of NAbs for antigenically distinct variants such as WT and Omicron because of the different optimal ranges of serum dilution. Potentially, this can lead to relatively low sensitivity in this test. In the present study, while pVNA showed a 16/9-fold increase for Sputnik V-/Salnavac-boosted donors (T2 to T1), respectively, the sVNT folds were only 1.5 for both vaccines, which, in general, was comparable to the previous study [73]. Testing the inhibition of RBD-ACE2 interactions by serial dilutions of serum may improve the sensitivity of chip-based sVNTs.

## 5. Conclusions

We would like to highlight that the COVID-19 pandemic triggered the rapid development of new vaccines and new methods of testing vaccine-induced immunity. Nasal vaccines appear attractive due to the induction of mucosal immunity, which is the first-line immune barrier [76]. Recently, it was shown that sIgA, in contrast to serum IgA, was able to neutralize new XBB and BA.2.86 Omicron subvariants after an Ad5-based Omicron vaccine booster [77]. Our results agree with this study in terms of the reduced breadth of humoral response to Omicron after a nasal booster. However, we showed that the muscular and nasal administration of the same vaccine induces specific serum antibodies with similar avidity, despite the lower level of nAbs after a nasal booster. These high-avidity antibodies circulated in the blood for a minimum of six months after the Salnavac or Sputnik V booster.

## Data Availability

The data that support the findings of this study are available from the corresponding author upon reasonable request.

## Supplementary Materials

The following supporting information can be downloaded at: https://www.mdpi.com/article/10.3390/vaccines12121362/s1, Figure S1: Effect of serum dilution on avidity indexes (AIs) for the RBD WT determined using ELISA with urea.

## References

[1] Plotkin S.A. (2010). Correlates of Protection Induced by Vaccination. Clin. Vaccine Immunol..

[2] Cromer D., Steain M., Reynaldi A., Schlub T.E., Wheatley A.K., Juno J.A., Kent S.J., Triccas J.A., Khoury D.S., Davenport M.P. (2022). Neutralising Antibody Titres as Predictors of Protection against SARS-CoV-2 Variants and the Impact of Boosting: A Meta-Analysis. Lancet Microbe.

[3] Mcdonald I., Murray S.M., Reynolds C.J., Altmann D.M., Boyton R.J. (2021). Comparative Systematic Review and Meta-Analysis of Reactogenicity, Immunogenicity and Efficacy of Vaccines against SARS-CoV-2. NPJ Vaccines.

[4] Victora G.D., Nussenzweig M.C. (2022). Germinal Centers. Annu. Rev. Ofimmunol..

[5] Rajewsky K. (1996). Clonal Selection and Learning in the Antibody System. Nature.

[6] Oostindie S.C., Lazar G.A., Schuurman J., Parren P.W.H.I. (2022). Avidity in Antibody Effector Functions and Biotherapeutic Drug Design. Nat. Rev..

[7] Doria-rose N.A., Joyce M.G. (2015). Strategies to Guide the Antibody Affinity Maturation Process. Curr. Opin. Virol..

[8] Polack F.P., Teng M.N., Collins P.L., Prince G.A., Exner M., Regele H., Lirman D.D., Rabold R., Hoffman S.J., Karp C.L. (2002). A Role for Immune Complexes in Enhanced Respiratory Syncytial Virus Disease. J. Exp. Med..

[9] Alexander M.R., Ringe R., Sanders R.W., Voss J.E., Moore J.P., Klasse J. (2015). What Do Chaotrope-Based Avidity Assays for Antibodies to HIV-1 Envelope Glycoproteins Measure?. J. Virol..

[10] Pullen G.R., Fitzgerald M.G., Hosking C.S. (1986). Antibody Avidity Determination by ELISA Using Thiocyanate Elution. J. Immunol. Methods.

[11] Khurana S., Verma N., Yewdell J.W., Hilbert A.K., Castellino F., Lattanzi M., Del Giudice G., Rappuoli R., Golding H. (2011). MF59 Adjuvant Enhances Diversity and Affinity of Antibody-Mediated Immune Response to Pandemic Influenza Vaccines. Sci. Transl. Med..

[12] Narita M., Matsuzono Y., Takekoshi Y., Yamada S., Itakura O., Kubota M., Kikuta H., Togashi T. (1998). Analysis of Mumps Vaccine Failure by Means of Avidity Testing for Mumps Virus-Specific Immunoglobulin G. Clin. Diagn. Lab. Immunol..

[13] Tsuji I., Dominguez D., Egan M.A., Dean H.J. (2022). Development of a Novel Assay to Assess the Avidity of Dengue Virus-Specific Antibodies Elicited in Response to a Tetravalent Dengue Vaccine. J. Infect. Dis..

[14] Ravichandran S., Hahn M., Belaunzarán-Zamudio P.F., Ramos-Castañeda J., Nájera-Cancino G., Caballero-Sosa S., Navarro-fuentes K.R., Ruiz-Palacios G., Golding H., Beigel J.H. (2019). Differential Human Antibody Repertoires Following Zika Infection and the Implications for Serodiagnostics and Disease Outcome. Nat. Commun..

[15] Monsalvo A.C., Batalle J.P., Lopez M.F., Krause J.C., Klemenc J., Hernandez J.Z., Maskin B., Bugna J., Rubinstein C., Aguilar L. (2011). Severe Pandemic 2009 H1N1 Influenza Disease Due to Pathogenic Immune Complexes. Nat. Med..

[16] Tang J., Ravichandran S., Lee Y., Grubbs G., Coyle E.M., Klenow L., Genser H., Golding H., Khurana S. (2021). Antibody Affinity Maturation and Plasma IgA Associate with Clinical Outcome in Hospitalized COVID-19 Patients. Nat. Commun..

[17] Hendriks J., Schasfoort R., Koerselman M., Dannenberg M., Cornet A.D., Beishuizen A., van der Palen J., Krabbe J., Mulder A.H.L., Karperien M. (2022). High Titers of Low Affinity Antibodies in COVID-19 Patients Are Associated with Disease Severity. Front. Immunol..

[18] Bauer G. (2021). The Potential Significance of High Avidity Immunoglobulin G (IgG) for Protective Immunity towards SARS-CoV-2. Int. J. Infect. Dis..

[19] Pušnik J., König J., Mai K., Richter E., Zorn J., Proksch H., Schulte B., Alter G., Streeck H. (2022). Persistent Maintenance of Atypical Memory B Cells Following SARS-CoV-2 Infection and Vaccination Recall Response. SSRN Electron. J..

[20] Infection S.-, Tauzin A., Gendron-lepage G., Nayrac M., Anand S.P., Bourassa C., Medjahed H., Goyette G., Dub M., Kaufmann D.E. (2022). Evolution of Anti-RBD IgG Avidity Following SARS-CoV-2 Infection. Viruses.

[21] Bullock J.L., Hickey T.E., Kemp T.J., Metz J., Loftus S., Haynesworth K., Castro N., Luke B.T., Lowy D.R., Pinto L.A. (2024). Longitudinal Assessment of BNT162b2- and mRNA-1273-Induced Anti-SARS-CoV-2 Spike IgG Levels and Avidity Following Three Doses of Vaccination. Vaccines.

[22] Scheiblauer H., Micha C., Wolf T., Khodamoradi Y., Bellinghausen C., Sonntagbauer M., Esser-nobis K., Filomena A., Mahler V., Jürgen T. (2022). Antibody Response to SARS-CoV-2 for More than One Year − Kinetics and Persistence of Detection Are Predominantly Determined by Avidity Progression and Test Design. J. Clin. Virol..

[23] Tauzin A., Gong S.Y., Chatterjee D., Ding S., Painter M.M., Goel R.R., Beaudoin-Bussières G., Marchitto L., Boutin M., Laumaea A. (2022). A Boost with SARS-CoV-2 BNT162b2 mRNA Vaccine Elicits Strong Humoral Responses Independently of the Interval between the First Two Doses. Cell Rep..

[24] Kashte S., Gulbake A., El S.F., Iii A., Gupta A. (2021). COVID-19 Vaccines: Rapid Development, Implications, Challenges and Future Prospects Indian Council of Medical Research. Hum. Cell.

[25] Krammer B.F., Ellebedy A.H. (2023). Variant-Adapted COVID-19 Booster Vaccines. Science.

[26] Nicolas A., Sannier G., Dubé M., Nayrac M., Tauzin A., Painter M.M., Goel R.R., Laporte M., Gendron-Lepage G., Medjahed H. (2023). An Extended SARS-CoV-2 MRNA Vaccine Prime-Boost Interval Enhances B Cell Immunity with Limited Impact on T Cells. iScience.

[27] Payne R.P., Longet S., Austin J.A., Skelly D.T., Dejnirattisai W., Adele S., Meardon N., Faustini S., Al-Taei S., Moore S.C. (2021). Immunogenicity of Standard and Extended Dosing Intervals of BNT162b2 mRNA Vaccine. Cell.

[28] Lavelle E.C., Ward R.W. (2022). Mucosal Vaccines—Fortifying the Frontiers. Nat. Rev. Immunol..

[29] Pilapitiya D., Wheatley A.K., Tan H. (2023). Review Mucosal Vaccines for SARS-CoV-2: Triumph of Hope over Experience. eBioMedicine.

[30] Van Doremalen N., Purushotham J.N., Schulz J.E., Holbrook M.G., Bushmaker T., Carmody A., Port J.R., Yinda C.K., Okumura A., Saturday G. (2021). Intranasal ChAdOx1 NCoV-19/AZD1222 Vaccination Reduces Viral Shedding after SARS-CoV-2 D614G Challenge in Preclinical Models. Sci. Transl. Med..

[31] Hassan A.O., Shrihari S., Gorman M.J., Curiel D.T., Alter G., Diamond M.S., Hassan A.O., Shrihari S., Gorman M.J., Ying B. (2021). Article An Intranasal Vaccine Durably Protects against SARS-CoV-2 Variants in Mice Ll An Intranasal Vaccine Durably Protects against SARS-CoV-2 Variants in Mice. Cell Rep..

[32] Zuev E.V., Markova O.A., Kulemzin S.V., Poteryaev D.A., Litvinova N.A., Korotkevich I.A., Grigoryeva T.V., Khamitov R.A. (2023). Virus Neutralizing Antibodies in Pseudovirus Particle Neutralization Reaction as a Bioanalytical Part of a Salnavac® Vaccine Clinical Trial. Russ. J. Infect. Immun..

[33] Wu S., Huang J., Zhang Z., Wu J., Zhang J., Hu H., Zhu T., Zhang J., Luo L., Fan P. (2021). Safety, Tolerability, and Immunogenicity of an Aerosolised Adenovirus Type-5 Vector-Based COVID-19 Vaccine (Ad5-NCoV) in Adults: Preliminary Report of an Open-Label and Randomised Phase 1 Clinical Trial. Lancet Infect. Dis..

[34] Aase A., Næss L.M., Sandin R.H., Herstad T.K., Oftung F., Holst J., Haugen I.L., Høiby E.A., Michaelsen T.E. (2003). Comparison of Functional Immune Responses in Humans after Intranasal and Intramuscular Immunisations with Outer Membrane Vesicle Vaccines against Group B Meningococcal Disease. Vaccine.

[35] Rothen D.A., Krenger P.S., Nonic A., Balke I., Vogt C.S., Chang X., Manenti A., Vedovi F., Resevica G., Walton S.M. (2022). Intranasal Administration of a Virus like Particles- Based Vaccine Induces Neutralizing Antibodies against SARS-CoV 2 and Variants of Concern. Allergy.

[36] Nguyen K.G., Mantooth S.M., Vrabel M.R., Zaharoff D.A. (2022). Intranasal Delivery of Thermostable Subunit Vaccine for Cross-Reactive Mucosal and Systemic Antibody Responses Against SARS-CoV-2. Front. Immunol..

[37] Zhang Z., Shen Q., Chang H. (2022). Vaccines for COVID-19: A Systematic Review of Immunogenicity, Current Development, and Future Prospects. Front. Immunol..

[38] Chavda V.P., Bezbaruah R., Valu D., Patel B., Kumar A., Prasad S., Kakoti B.B., Kaushik A., Jesawadawala M. (2023). Adenoviral Vector-Based Vaccine Platform for COVID-19: Current Status. Vaccine.

[39] Vanaparthy R., Mohan G., Vasireddy D., Atluri P. (2021). Review of COVID-19 Viral Vector-Based Vaccines and COVID-19 Variants. Le. Infez. Med..

[40] Bellusci L., Grubbs G., Zahra F.T., Forgacs D., Golding H., Ross T.M., Khurana S. (2022). Antibody Affinity and Cross-Variant Neutralization of SARS-CoV-2 Omicron BA.1, BA.2 and BA.3 Following Third mRNA Vaccination. Nat. Commun..

[41] Singh G., Abbad A., Tcheou J., Mendu D.R., Firpo-Betancourt A., Gleason C., Srivastava K., Cordon-Cardo C., Simon V., Krammer F. (2023). Binding and Avidity Signatures of Polyclonal Sera From Individuals With Different Exposure Histories to Severe Acute Respiratory Syndrome Coronavirus 2 Infection, Vaccination, and Omicron Breakthrough Infections. J. Infect. Dis..

[42] Fiedler S., Brugger S.D., Von A., Emmenegger M., Fiedler S., Brugger S.D., Devenish S.R.A., Morgunov A.S., Meisl G., Lynn A.K. (2022). Both COVID-19 Infection and Vaccination Induce High-Affinity Cross-Clade Responses to SARS-CoV-2 Variants. iScience.

[43] Gorchakov A.A., Kulemzin S.V., Guselnikov S.V., Baranov K.O., Belovezhets T.N., Mechetina L.V., Volkova O.Y., Najakshin A.M., Chikaev N.A., Chikaev A.N. (2021). Isolation of a Panel of Ultra-Potent Human Antibodies Neutralizing SARS-CoV-2 and Viral Variants of Concern. Cell Discov..

[44] Khurana S., Hahn M., Coyle E.M., King L.R., Lin T., Treanor J., Sant A., Golding H. (2019). Repeat Vaccination Reduces Antibody Affinity Maturation across Different Influenza Vaccine Platforms in Humans. Nat. Commun..

[45] Alu A., Chen L., Lei H., Wei Y., Tian X., Wei X. (2022). Intranasal COVID-19 Vaccines: From Bench to Bed. eBioMedicine.

[46] Hao Y., Yang H.S., Karbaschi M., Racine-Brzostek S.E., Li P., Zuk R., Yang Y.J., Klasse P.J., Shi Y., Zhao Z. (2022). Measurements of SARS-CoV-2 Antibody Dissociation Rate Constant by Chaotrope-Free Biolayer Interferometry in Serum of COVID-19 Convalescent Patients. Biosens. Bioelectron..

[47] Dimitrov J.D., Lacroix-Desmazes S., Kaveri S.V. (2011). Important Parameters for Evaluation of Antibody Avidity by Immunosorbent Assay. Anal. Biochem..

[48] Eltanbouly M.A., Ramos V., Maclean A.J., Chen S.T., Loewe M., Steinbach S., Tanfous T.B., Johnson B., Cipolla M., Gazumyan A. (2024). Role of Affinity in Plasma Cell Development in the Germinal Center Light Zone. J. Exp. Med..

[49] Nguyen D.C., Hentenaar I.T., Morrison-Porter A., Solano D., Haddad N.S., Castrillon C., Runnstrom M.C., Lamothe P.A., Andrews J., Roberts D. (2024). SARS-CoV-2-Specific Plasma Cells Are Not Durably Established in the Bone Marrow Long-Lived Compartment after mRNA Vaccination. Nat. Med..

[50] Klasse P.J. (2016). How to Assess the Binding Strength of Antibodies Elicited by Vaccination against HIV and Other Viruses. Expert. Rev. Vaccines.

[51] Li K., Dodds M., Spreng R.L., Abraha M., Huntwork R.H.C., Dahora L.C., Nyanhete T., Dutta S., Wille-Reece U., Jongert E. (2023). A Tool for Evaluating Heterogeneity in Avidity of Polyclonal Antibodies. Front. Immunol..

[52] Brady A.M., Unger E.R., Panicker G. (2017). Description of a Novel Multiplex Avidity Assay for Evaluating HPV Antibodies. J. Immunol. Methods.

[53] Polack F.P., Hoffman S.J., Crujeiras G., Griffin D.E. (2003). A Role for Nonprotective Complement-Fixing Antibodies with Low Avidity for Measles Virus in Atypical Measles. Nat. Med..

[54] Portilho A.I., Santos J.S., Trzewikoswki de Lima G., Lima G.G., De Gaspari E. (2023). Study of Avidity-ELISA: Comparison of Chaotropic Agents, Incubation Temperature and Affinity Maturation after Meningococcal Immunization. J. Immunol. Methods.

[55] Correa V.A., Rodrigues T.S., Portilho A.I., Trzewikoswki de Lima G., De Gaspari E. (2021). Modified ELISA for Antibody Avidity Evaluation: The Need for Standardization. Biomed. J..

[56] Hollander Z., Katchalski-Katzir E. (1986). Use of Monoclonal Antibodies to Detect Conformational Alterations in Lactate Dehydrogenase Isoenzyme 5 on Heat Denaturation and on Adsorption to Polystyrene Plates. Mol. Immunol..

[57] Butler J.E., Navarro P., Sun J. (1997). Adsorption-induced antigenic changes and their significance in ELISA and immunological disorders. Immunological and Molecular Diagnosis of Infectious Disease.

[58] Butler J.E., Ni L., Nessler R., Joshi K.S., Suter M., Rosenberg B., Chang J., Brown W.R., Cantarero L.A. (1992). The physical and functional behavior of capture antibodies adsorbed on polystyrene. J. Immunol. Methods..

[59] Kwong J.C., Chung H., Jung J.K.H., Buchan S.A., Campigotto A., Campitelli M.A., Crowcroft N.S., Gubbay J.B., Karnauchow T., Katz K. (2020). The Impact of Repeated Vaccination Using 10-Year Vaccination History on Protection against Influenza in Older Adults: A Test-Negative Design Study across the 2010/11 to 2015/16 Influenza Seasons in Ontario, Canada. Eurosurveillance.

[60] Baumgarth N. (2013). How Specific Is Too Specific? B-Cell Responses to Viral Infections Reveal the Importance of Breadth over Depth. Immunol. Rev..

[61] Horndler L., Delgado P., Romeropinedo S., Llamas M.A., Almendrova P. (2023). Decreased Breadth of the Antibody Response to the Spike Protein of SARS-CoV-2 after Repeated Vaccination. Front. Immunol..

[62] Astakhova E.A., Morozov A.A., Byazrova M.G., Sukhova M.M., Mikhailov A.A., Minnegalieva A.R., Gorchakov A.A., Filatov A.V. (2023). Antigenic Cartography Indicates That the Omicron BA.1 and BA.4/BA.5 Variants Remain Antigenically Distant to Ancestral SARS-CoV-2 after Sputnik V Vaccination Followed by Homologous (Sputnik V) or Heterologous (Comirnaty) Revaccination. Int. J. Mol. Sci..

[63] Edara V.V., Manning K.E., Ellis M., Lai L., Moore K.M., Foster S.L., Floyd K., Davis-Gardner M.E., Mantus G., Nyhoff L.E. (2022). mRNA-1273 and BNT162b2 mRNA Vaccines Have Reduced Neutralizing Activity against the SARS-CoV-2 Omicron Variant. Cell Rep. Med..

[64] Muecksch F., Weisblum Y., Barnes C.O., Bjorkman P.J., Hatziioannou T., Bieniasz P.D., Muecksch F., Weisblum Y., Barnes C.O., Schmidt F. (2021). Article Affinity Maturation of SARS-CoV-2 Neutralizing Antibodies Confers Potency, Breadth, and Resilience to Viral Escape Mutations Ll Affinity Maturation of SARS-CoV-2 Neutralizing Antibodies Confers Potency, Breadth, and Resilience to Viral Escape. Immunity.

[65] Huang C.Q., Vishwanath S., Carnell G.W., Chun A., Chan Y., Heeney J.L. (2023). Immune Imprinting and Next-Generation Coronavirus Vaccines. Nat. Microbiol..

[66] Nakagama Y., Candray K., Kaku N., Komase Y., Rodriguez-Funes M.V., Dominguez R., Tsuchida T., Kunishima H., Nagai E., Adachi E. (2023). Antibody Avidity Maturation Following Recovery From Infection or the Booster Vaccination Grants Breadth of SARS-CoV-2 Neutralizing Capacity. J. Infect. Dis..

[67] Tan C.W., Chia W.N., Qin X., Liu P., Chen M.I.C., Tiu C., Hu Z., Chen V.C.W., Young B.E., Sia W.R. (2020). A SARS-CoV-2 Surrogate Virus Neutralization Test Based on Antibody-Mediated Blockage of ACE2–Spike Protein–Protein Interaction. Nat. Biotechnol..

[68] Mariën J., Michiels J., Heyndrickx L., Nkuba-Ndaye A., Ceulemans A., Bartholomeeusen K., Madinga J., Mbala-Kingebeni P., Vanlerberghe V., Ahuka-Mundeke S. (2021). Evaluation of a Surrogate Virus Neutralization Test for High-Throughput Serosurveillance of SARS-CoV-2. J. Virol. Methods.

[69] Santos da Silva E., Servais J.Y., Kohnen M., Arendt V., Staub T., Krüger R., Fagherazzi G., Wilmes P., Hübschen J.M., Ollert M. (2023). Validation of a SARS-CoV-2 Surrogate Neutralization Test Detecting Neutralizing Antibodies against the Major Variants of Concern. Int. J. Mol. Sci..

[70] Kanokudom S., Assawakosri S., Suntronwong N., Auphimai C., Nilyanimit P., Vichaiwattana P., Thongmee T., Yorsaeng R., Srimuan D., Thatsanatorn T. (2022). Safety and Immunogenicity of the Third Booster Dose with Inactivated, Viral Vector, and mRNA COVID-19 Vaccines in Fully Immunized Healthy Adults with Inactivated Vaccine. Vaccines.

[71] Kim S.J., Yao Z., Marsh M.C., Eckert D.M., Kay M.S., Lyakisheva A., Pasic M., Bansal A., Birnboim C., Jha P. (2022). Homogeneous Surrogate Virus Neutralization Assay to Rapidly Assess Neutralization Activity of Anti-SARS-CoV-2 Antibodies. Nat. Commun..

[72] Lynch K.L., Zhou S., Kaul R., Walker R., Wu A.H. (2022). Evaluation of Neutralizing Antibodies against SARS-CoV-2 Variants after Infection and Vaccination Using a Multiplexed Surrogate Virus Neutralization Test. Clin. Chem..

[73] Heggestad J.T., Britton R.J., Kinnamon D.S., Wall S.A., Joh D.Y., Hucknall A.M., Olson L.B., Anderson J.G., Mazur A., Wolfe C.R. (2021). Rapid Test to Assess the Escape of SARS-CoV-2 Variants of Concern. Sci. Adv..

[74] McDade T.W., Demonbreun A.R., Sancilio A., Mustanski B., D’Aquila R.T., McNally E.M. (2021). Durability of Antibody Response to Vaccination and Surrogate Neutralization of Emerging Variants Based on SARS-CoV-2 Exposure History. Sci. Rep..

[75] Springer D.N., Höltl E., Prüger K., Puchhammer-Stöckl E., Aberle J.H., Stiasny K., Weseslindtner L. (2024). Measuring Variant-Specific Neutralizing Antibody Profiles after Bivalent SARS-CoV-2 Vaccinations Using a Multivariant Surrogate Virus Neutralization Microarray. Vaccines.

[76] He X., Chen X., Wang H., Du G., Sun X. (2023). Recent Advances in Respiratory Immunization: A Focus on COVID-19 Vaccines. J. Control Release.

[77] Chen S., Zhang Z., Wang Q., Yang Q., Yin L., Ning L., Chen Z., Tang J., Deng W., He P. (2024). Intranasal Adenovirus-Vectored Omicron Vaccine Induced Nasal Immunoglobulin A Has Superior Neutralizing Potency than Serum Antibodies. Signal Transduct. Target. Ther..

