# Supplementary material for: Antibody Avidity Maturation Following Booster Vaccination with an Intranasal Adenovirus Salnavac Vaccine
Source: Vaccines (Basel). 2024 Dec 2;12(12):1362. doi: 10.3390/vaccines12121362 (PMC11680177; doi:10.3390/vaccines12121362)
Supplement: Supplementary file 1 [file vaccines-12-01362-s001.zip › vaccines-3282083-supplementary.pdf]

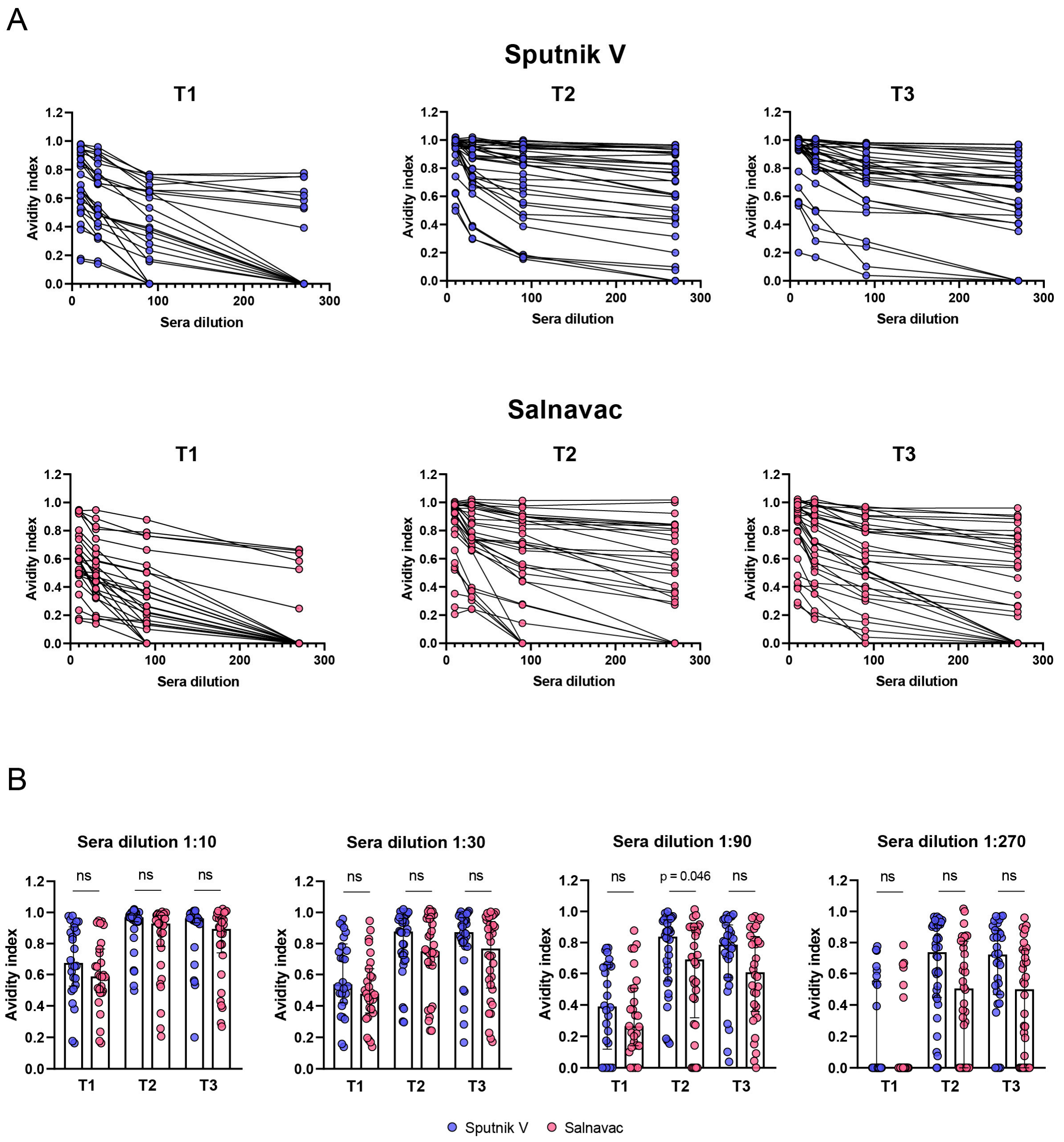

Figure S1. Effect of serum dilution on avidity indexes (AIs) for the RBD WT determined using ELISA with urea. (A) Changes in Ais of RBD-specific IgG sera antibodies from Sputnik V (top) and Salnavac-boosted (bottom) individuals at timepoints T1, T2, and T3. (B) Comparison of Ais of RBD-specific IgG sera antibodies from Sputnik V and Salnavac-boosted individuals. Statistics were calculated using Kruskal-Wallis test with Dunn's multiple comparisons test. P value is indicated (B); ns—non-significant differences.
